# Men, relationships and partner-initiated break-ups: A narrative analysis

**DOI:** 10.1177/20551029221142465

**Published:** 2022-11-26

**Authors:** John L Oliffe, Mary T Kelly, Gabriela Gonzalez Montaner, David Kealy, Zac E Seidler, John S Ogrodniczuk, Paul Sharp, Simon M Rice

**Affiliations:** 1 School of Nursing, 70439 University of British Columbia, Vancouver, BC, Canada; 2 Department of Nursing, University of Melbourne, Vancouver, BC, Canada; 3 Department of Psychiatry, 70439 University of British Columbia, Vancouver, BC, Canada; 4 Centre for Youth Mental Health, The University of Melbourne, Parkville, VIC, Australia

**Keywords:** Masculinity, men’s relationships, men’s mental health, domestic violence prevention, narrative analysis

## Abstract

For men, significant risks associated with partner-initiated break-ups include domestic violence, mental health challenges and difficultly with life transition. This narrative analysis study shares three storylines drawn from interviews with 25 men who experienced a partner-initiated break-up. Ill equipped to stay or to initiate leaving narratives positioned participants as conflict averse, lacking agency and withdrawing emotionally from the partnership and its demise. Victims of circumstance narratives included men who engaged in cyclic arguments and ongoing power struggles with partners, a pattern that often amplified conflict after the break-up. Transitioning these two impasse narratives were some participants whose Accountability and growth storylines highlighted their introspective self-work, aided by resources including professional help to deconstruct, understand, and adjust their behaviours. Making connections to masculinities theory, these findings suggest that tailored interventions, including narrative therapy, might usefully interrupt impasse narratives to aid men’s development and healthful transitions through partner-initiated break-ups.

## Introduction

Many men experience the end of an intimate partner relationship as injurious, and cope by relying on gendered practices that include the ineffectual concealment and/or expression of negative emotions and mental health challenges (Oliffe et al., 2022). The effects of relationship break-ups range from emotional distress with disruptions to men’s social status (Hartman, 2021) through self-harm and suicide as revenge for partner betrayal (River and Flood, 2021; Khan et al., 2021). Partner-initiated break-ups in particular can result in rejection sensitivities that heighten the potential for men to perpetrate intimate partner violence (IPV) and domestic violence (DV) (Downey et al., 2000) and in extreme cases, homicide (Kalish and Kimmel, 2010). Empirical insights delineating contributing factors and pathways for the aforementioned dire outcomes (i.e. IPV, DV, male suicide, homicide) have been helpful. However, insufficient research attention has been paid to men’s perspectives about partner-initiated break-ups as a means to thoughtfully consider how tailored interventions might garner upstream IPV and DV prevention and mental health promotion (Oliffe et al., 2022). The current study addresses this knowledge gap by making available three discrete yet interconnected narratives drawn from men who experienced a partner-initiated break-up.

## Masculinities, mental health and partner-initiated break-ups

Connell’s (2005) masculinities theory has been highly influential in research on men’s health and illness, and in the call for gender-specific and transformative social and mental health care services (Kågesten and Chandra-Mouli, 2020; Robertson et al., 2016; Seidler et al., 2018). Amid the plurality of masculinities operating in response to dominant masculine ideals (i.e. emotional stoicism, self-reliance, strength), the relational nature of gender (Connell and Messerschmidt, 2005) has featured in explanations connecting men’s mental illness challenges and disrupted intimate partner relationships (Oliffe et al., 2022). Herein, gender relations reveal co-constructed masculinities in varying social and structural contexts as strongly influencing men’s behaviours (Oliffe et al., 2011; Lyons, 2009). For example, in regard to partner-initiated break-ups, men are consistently depicted as brooding, solemn, risk-takers overusing alcohol and other drugs to wash away and distract their painful emotions (Esper and Furtado, 2013; Simon and Barrett, 2010). The other often-told and related narrative features men’s anger for all that was invested (and lost) in the relationship, including their sense of wasted emotional labour along with break-up induced financial losses and/or restricted access to their children (McQueen, 2017). Men’s anger as socially normed but deeply stigmatized is consistently linked to IPV and DV, as well as male self-harm and suicidality (River and Flood, 2021; Khan et al., 2021). Even though such dire outcomes occur in the minority of partner-initiated break-ups (Kerr and Capaldi, 2011), most storylines belabour the role of unhealthy masculinities characterized by domination, control, aggression and power as key drivers of men’s negative behaviours both within and following distressed intimate partner relationships.

Less newsworthy are the majority of partner-initiated break-ups where men’s challenging transitions are less violent. For example, work by Hartman (2021) indicated that partner-initiated break-ups can be powerful drivers for men denying and downplaying their sense of failure, weakness and loneliness with cushioning effects garnered by swiftly moving on to secure a new romantic interest. Also reported were how some men resist masculine ideals such as stoicism and conquest, preferring to sit with and process their emotions in the aftermath of their partner ending the relationship (Hartman, 2021). Hartman (2019), in a separate analysis of divorced fathers, clarified that men who did not express their emotions or share details of the separation with their children, did so to shield their children, and ensure ongoing contact with them. In this specific context, protection and provider identities, as idealized masculinities, might be argued as strength-based efforts for reducing conflict within disrupted intimate partner relationships (Hartman, 2019). Hartman’s (2019, 2021) work also depicted participants as relying on, rejecting, or reformulating masculine ideals to transition and recover from partner-initiated break-ups in non-violent ways. The aforementioned research on masculinities, men’s mental health challenges and intimate partner relationships can be conceptualized as somewhat awkwardly occupying a deficit - strength binary. That is, deficit models consistently connect patriarchy to men’s power plays, dominance, control and aggression in and after relationship break-ups (Connell and Messershmidt, 2005; River and Flood, 2021; Khan et al., 2021); whereas strength-based masculinities highlight men who embody efforts for self-health, and the well-being of significant others (Hartman, 2019, 2021; Rice et al., 2021).

Of course, partner-initiated break-ups lever diverse masculine practices across a deficit - strength continuum. While masculinities research has reported men’s mental health behaviours and illness experiences (Emslie et al., 2006), narrative analyses can offer additional insights to how participants subjectively perceive and depict themselves, and others, within and across significant life events – including relationships and partner-initiated break-ups (Sahpazi and Balamoutsou, 2015). For example, Doering’s (2010) mixed-sex sample narrative study indicated that partner-initiated break-ups were associated with the person who was left saving face by denying injury, emphasizing agency, and pointing out valuable self-changes. Narrative analyses have also revealed storytellers’ masculine practices to guide the use of interventions, including narrative therapy, that aim to question and re-shape men’s stories, schemas and understandings. Indeed, narrative therapy has been successfully used with men who have abused partners (Augusta-Scott, 2009); however, it remains an under-utilized resource for men building their relationship skills and mental health. The current study extends narrative research by making available insights to the gendered dimensions of men’s stories about relationships and partner-initiated break-ups to thoughtfully consider how tailored interventions might be used in upstream IPV and DV prevention and mental health promotion programs.

## Methodology

A narrative methodology was employed that recognized men’s stories about relationships and partner-initiated break-ups as authentic expressions of their internal states and beliefs (Squire et al., 2013). Comprising a constructionist approach, participants’ stories were understood as changing across time (Esin et al., 2014), and the focus was on how men positioned themselves and partners in their narratives, paying attention to evolutions, contradictions and points of emphasis (Holstein and Gubrium, 2013). Keenly interested to understand how narratives operate dialogically between the personal and the surrounding social worlds that produce, consume, silence and contest them (Davies and Harré, 1990), Connell’s (2005) masculinities framework was used to interpret participants’ metaphors, storylines and concepts.

### Study criteria and recruitment

This narrative analysis focussed on men who had experienced a partner-initiated break-up, a sub-group harvested from a larger study that included participants who had left or amicably ended intimate partner relationships (Oliffe et al., In press, 2022). Following ethics approval from the University of British Columbia (ID: H20-01868), participants were recruited via Twitter, Facebook, Reddit and targeted emails inviting men who had experienced an intimate partner relationship break-up to complete an individual in-depth Zoom interview to share their story (Oliffe et al., 2022). Potential participants contacted the project manager via email, and eligibility was assessed (i.e. male, living in Australia or Canada, ≥19 years, past experience of a relationship break-up, and English speaking) ahead of providing a link to complete an e-consent and demographics questionnaire including the Patient Health Questionnaire (PHQ-9), a brief scale that screens for depression symptoms and includes a single item to assess suicidal ideation (Kroenke et al., 2001). Once consent was obtained, one of 4 researchers (2 females and 2 males based in Canada) scheduled a 60-min semi-structured interview. Participants were sent a $100 e-gift card after the interview in recognition of their time and contribution to the study.

### Participants and data collection

Twenty-five men who had experienced a partner-initiated break-up took part in the study. Participants comprised Canadian (*n* = 13; 52%) and Australian-based (*n* = 12; 48%) men ranging in age from 26 to 64 years (M = 41.96; SD = 9.40) who self-identified as heterosexual (*n* = 22; 88%), bisexual (*n* = 2; 8%), and gay (*n* = 1; 4%). Over half of the men were fathers (*n* = 17; 68%) and 16 (64%) were separated/divorced and single at the time of the interview (Please see Table 1 Participant Demographics). Zoom interviews took place from June 2020 to February 2022, and questions included, ‘what was your role in the relationship and the break-up?‘, ‘what were the main causes of the relationship ending?‘, ‘what emotions did you experience and express in the relationship, and after the break-up?’

**Table 1.** Participant demographics (*n* = 25).

Age (years) (Range 26–64; Mean 41.96)	*n* (%)
20–29	2 [8]
30–39	6 [24]
40–49	14 [56]
50–59	1 [4]
60–69	2 [8]
**Gender**	
Male	24 [96]
Gender queer/Gender non-binary	1 [4]
**Sexuality**	
Heterosexual	22 [88]
Bisexual	2 [8]
Gay	1 [4]
**Education (highest level completed)**	
Some high school	3 [12]
Some college	3 [12]
Diploma or certificate	5 [20]
Bachelor’s degree	10 [40]
Postgraduate degree	4 [16]
**Marital status**	
Single, previously separated or divorced	16 [64]
Partnered or married [previously dated/common-law/divorced]	5 [20]
Single, never married	4 [16]
**Current living arrangements**	
Lives with children	9 [36]
Lives alone	7 [28]
Other family member(s) (e.g., parents)	4 [16]
Partner/spouse and child (ren)	3 [12]
Roommates	2 [8]
**Fathers**	
Fathers	17 [68]
**Who do you talk to about your relationship? (Check all that apply)**	
Friend(s)	19 [76]
Healthcare professional	16 [64]
Family	15 [60]
Partner/spouse/new partner	9 [36]
Facilitated peer group	4 [16]
**PHQ-9**	
None-minimal [0–4]	9 [36]
Mild [5–9]	7 [28]
Moderate [10–14]	3 [12]
Moderately severe [15–19]	5 [20]
Severe [20–27]	1 [4]
**Suicidal ideation in past 2 weeks**	
Yes	7 [28]
No	18 [72]

### Data analysis

Field notes about the interview situation and interactions including features of the conversation such as crying, laughing, pauses, tone and pitch were added to each transcribed interview (Frost, 2009). Three interviewers and authors (JLO, MTK, GGM) immersed themselves in the data, reading and re-reading the transcripts and listening to the recorded interviews to make written summaries inclusive of each participant’s specific context and overarching storyline. Analyses progressed by focussing on participant narratives to address the research question, *how do men position themselves within the relationship and after the partner-initiated break-up*? Memos were made to document and develop interpretations for why each participant told the story the way they did, amid beginning to iteratively define and assign a narrative label to each participant. These analyses developed with fortnightly researcher meetings for 18 months concurrent with data collection. Each interview, as a unit of analysis, was discussed and categorized in selecting narrative segments as micro units for fine-grained analyses, and determining illustrative quotes to be shared as representative of specific storylines (Riessman, 1993).

Based on what predominated in each man’s storyline, participants were assigned to one of three inductively derived narratives; 1) Ill-equipped to stay or to initiate leaving, 2) Victims of circumstance, and 3) Accountability and growth. The first two narratives were impasse storylines, wherein participants *Ill-equipped to stay or to initiate leaving* and *Victims of circumstance* stories positioned them as somewhat stuck, without resources for successfully correcting the relationship or transitioning the break-up. The *Accountability and growth* narratives, by contrast, depicted some participant’s commitment to, and strategies for transitioning the two impasse storylines to chronicle their introspective self-work in adjusting their behaviours. Writing was central to these analyses, and dialogical questions including, *who does the story connect the storyteller to?* and *who is placed outside those connections?* (Frank, 2012) were addressed to advance the findings. In revisiting the participant interviews allocated to each of the three narratives, Connell’s (2005) masculinities framework was used to interpret and report the gendered dimensions of the men’s storylines.

## Results

### Narrative 1. ill-equipped to stay or to initiate leaving

Evident across 10 men’s interviews were *Ill-equipped to stay or to initiate leaving* narratives in which participants characterized themselves as lacking the agency to effectively address problems that arose, or decisively work toward ending distressed partnerships. These men seemingly accepted the fate of their relationship as being in limbo, arraigning themselves as subordinate and passive in the partnership, unable to influence their stalled and sub-optimal relationship. For some participants, including 31-year-old Justin, troublesome dynamics and his abeyance for addressing them, were long-standing. He began his story by explaining that he was initially rejected by his partner, and though he was pleasantly surprised that she eventually changed her mind, Justin harboured doubts that her feelings for him matched his passionate interests for her:

“Like basically I had chased her around all summer just to have her say ‘no’, so it got to a point where it was like ‘okay I can’t really do much about it’. And then when the second year of university started, all of a sudden she was expressing quite a bit of interest…I think we were at the bar for a party and she said she was kind of drunk and just felt an irresistible urge to kiss me, so not really sure where it came from…the cynical part of me is, like I know she had relations with my roommate in the first year and I think he had rejected her pretty early on. I don’t want to speculate, right but it’s easy to look at it as I was kind of second choice and was just there, right.”

From the outset Justin evaluated himself as lesser in terms of influence on and agency in the relationship, wherein his partner ultimately decided on the connection she had earlier spurned – a trend that carried through to her eventually breaking off their 3-year partnership. Justin’s doubts manifested concerns that he felt too strongly for his partner. He talked about wanting to have sex more often than his partner, and his resentment for going to her work functions or socializing with her friends amid mocking her dreams of being an Olympian. These gripes flared with his partner’s career growth and her new and burgeoning friendships and interests, the sum of which heightened Justin’s insecurities and uncertainty for how to comfortably be in the relationship:

“If I let her just go do her own thing, then I was basically getting shoved off to the side, and if I put my foot down and said like ‘no, if you care at all, right, you’ll come up [to visit]’, then I’d be controlling.”

Though Justin agonized over his carefree - control bind, he steadfastly avoided discussing this quandary (and vulnerability) with his partner. He explained “my dad is still that way [controlling]” in suggesting that he was trying to “reprogram myself because I agree it’s problematic.” Here, the flaws for craving control wedged in that Justin knew he was reproducing outdated and stigmatized masculine practices. Justin also suggested that his partner’s lack of gratitude and reciprocity for what he did and felt intensified the pain of his muted resentments:

“I did do a lot of things for her as opposed to for me, but also under the guise of well, ‘if I’m doing things for her, I’m also doing things for me’, which was a lot of the friction. I wasn’t acknowledging what I wanted and what I needed because at the end of the day I was afraid of losing out on my first serious relationship.”

Justin’s emotional stoicism might be understood as reflecting alignments to masculinities idealized by wanting to appear rational, self-assured, unemotional and strong in and for the relationship. However, contrasting those idealized embodiments were Justin’s vulnerability narratives, a storyline that carried on long after the break-up, “basically the conclusion that I’ve come to…I think that if I would have catered to her even more than I had already done, it wouldn’t have changed a thing.”

Many men signaled their lack of agency as relational in which their silences and withdrawal were connected to their partner’s distress and dominance. In these depictions participants portrayed themselves as forever conceding within and/or retreating from the relationship to avoid conflict. Cody, a 49-year-old father said, “her [partner] needs were so strong and so prevalent that I couldn’t get an edge in there” in summating their 20-year relationship. Cody’s disinterest and detached standpoint was positioned as easing the powerlessness he felt in his subordinate state:

“I wasn’t terribly sensitive about what she was going through [family, career and mental health issues] because I didn’t understand it, and I didn’t make many attempts to do so.”

Yet Cody’s detachment was also self-protective in trying to sustain what had effectively become a shared house and platonic roommate arrangement:

“I always vehemently denied it [unhappiness] because the prospect [breaking-up] of what that would entail if I did admit to it, was way too great. That would mean a loss of everything and I wasn’t prepared to do that. So it took her to take the initiative and make the step [break-up]. I don’t know whether to thank her now or not because it has been a tough ride.”

An ambivalence-based withdrawal underpinned Cody’s narrative, wherein he situated himself as estranged but unwilling to end the partnership amid feeling powerless to curb his partner’s dominance and actions for closing their relationship. Similarly, Billy, a 48-year-old father, explained that in his 15-year marriage it had “become a physical anxiety to talk about things” because you’re “too scared” and “fear saying something.” These conflict avoidant states allowed men to stay in the relationship, though they narrated their dissatisfaction with the ill-state of the partnership. These narrative builds also revealed an awareness (and resignate masculinity) that their in-relation subordinate status would carry through to their partner initiating the break-up.

In addition, there were some failed pleaser efforts in which participants talked about being unable to meet their partner’s expectations. Samuel, a 42-year-old father who was married for 20-years contrasted how his wife “was a very go and do it type…where I was a little bit more laid back in my ways of dealing with things” as a fundamental, but eventually relationship-ending difference:

“I felt like I was doing everything she wanted me to do [as a father] but I just couldn’t grow the way that she would probably want me to grow. And I felt guilty, I felt like I failed.”

Samuel contrasted happier times when he and his wife “worked together pretty well in those eight years before kids” concluding that his slide was sealed by his parenting shortfalls, to the extent that his partner ended the relationship. Phil, a 35-year-old said, “the first six months were fine and then for about 2 years after that, there was always this reoccurring discussion of her pursuing, meaning, “‘we're not being intimate enough…I'm curious about exploring other people.’” In these contexts, participants conceded their underperformance in the relationship (i.e., parenting, libido) in accepting their partner’s grievances. Moreover, these men depicted themselves as unable to improve or satisfy their partner to save the relationship.

Though most men indicated that they expected to be left, there were intense and painful emotions when the relationship ended. Samuel, for example, tearfully recounted his break-up:

“When the day comes when she [partner] says that she doesn't want you to sleep next to her anymore, that hurt a lot too and it felt like she was pushing me away when that happened. She had suggested it and I did it as respect. So that was very hard to deal with as a man and…I just felt like I couldn't face the storm so to speak…I said to her, ‘look, I'm here’. She goes ‘I know you're here, but you are emotionally not here’.”

Samuel narrated his inability to slow or prevent the break-up, conceding his partner was right – in that he was emotionally inept and unable to meet her needs. Yet, Samuel lingered because he recognized himself as incapable for coping with the end of the partnership. Like Samuel, Cody’s story acknowledged his distress in the relationship, but nonetheless the end of the partnership was excruciatingly painful:

“Mass confusion is probably the biggest feeling that I had. I didn’t understand what was going on. And possibly some depression, actually, no, definitely some depression going on in there too. Depression like based off of my whole world was coming to a close, it was falling apart. I think I lost my job at the same time.”

Narrating the pain of the partnership ending, Cody starkly contrasted his ambivalence and withdrawal narrative for how he was in the relationship. This shifting storyline did however consistently highlight Cody’s silences throughout the layering subordinate effects in and after the partnership.

Men’s Ill-equipped to stay or to initiate leaving narratives revealed how emotional stoicism, withdrawal, and/or failed pleaser efforts rendered them bystanders in their relationship, and its end. Subordinate, participants consistently story-lined their lack of agency and relationship skills as a resignate masculinity in which their partner would inevitably initiate the break-up.

### Narrative 2: victims of circumstance

Ten participants told *Victims of circumstance* narratives depicting themselves as active agents contributing to conflict-ridden relationships that culminated in injurious partner-initiated break-ups. These narratives were often foregrounded and contrasted with details about happier times, in ways that underscored the jolt of finding themselves locked into palpably troubled partnerships. Brad, a 52-year-old man, led with, “when the divorce happened it nearly destroyed me” ahead of telling the backstory to his 14-year relationship. He explained, somewhat coyly, that their partnership happened “almost by chance”, wryly recounting, “her TV was broken, I fixed her TV. And then, you know, one thing led to another.” Brad talked about his partner’s beauty, and their smitten fun-filled courtship cementing the relationship:

“On a physical level, she was very striking, dark hair, blue eyes…she had a British accent, which I also find really attractive…we started to visit the Greek islands and then suddenly it was like, wow, this is so great. So it's quite a lovely, literally a honeymoon kind of experience. So we had lots of fun traveling around together and then we traveled around in Europe together too.”

Contrasting his attraction and their happiness, unending arguments grew to dominate and define the relationship; Brad shared numerous examples in suggesting that he was ultimately victim to his partner’s relentless efforts to dominate him, and their partnership:

“I realized that I was the one who was apologizing after a while on almost a daily basis…I can count maybe on one hand the number of times that she [partner] actually apologized in our entire 14 years together. So she wasn’t someone who could back down and apologize easily. So kind of a tough, I don’t want to draw too many analogies to Margaret Thatcher but she was a tough one. Let’s just say she was a tough woman.”

The lilt and lure of his partner’s accent and beauty had waned, and Brad’s gendered references turned his early (and implicitly naive) attraction to the trouble ignited by her uncompromising “Iron Lady” affects. He quipped about his failed “Nightingale syndrome” efforts to “heal her heart and open her heart” narrating various triggers including the “financial tribulations” of purchasing a defective condominium, parenting two young children, and the death of their own parents. Brad’s storyline also built to position his job loss as the final lever for his partner’s merciless decision to end their relationship:

“I said, you know, ‘if someone falls into the mud, if you can’t help them, at least leave them alone, don’t go over there and kick them in the head some more or stamp on them or crush them into the mud’. But that’s what I felt like she was doing. She was just continuing to hammer me and smash me.”

After losing his job, and bundling the tensions that flowed from his disgruntlement for being a stay-at-home dad with the exacerbation of a lifelong (but previously well-managed) depression, Brad narrated and normed defending himself, “I can be a fiery character… lacking emotional control sometimes…So, the relationship has been, you know, fiery.” Engaged with ever-present conflict, many men similarly spoke of tensions characterizing their partnership and its demise. Levi, a 31-year-old, regretted his 2-year partnership, summating that, “it was actually chaos, like yelling at each other, fighting, swearing, really mean things were said, and it just wasn’t a good relationship.” The participant position in these narratives was one in which they rationally expressed and defended themselves in the relationship but were ultimately victims of circumstance in that they could not secure a win, concede defeat, or negotiate a cease fire to sustain the partnership.

Some men also depicted themselves as victims by overcompensating in their efforts to reduce tensions in the relationship. Charles, a 42-year-old man, foregrounded his anxiety and depression as pressure-points in narrating his compromise to recompense his partner for their sexless marriage:

“So we’d gone 10 years without having sex…I remember at the 10-year mark we actually tried to have sex and then we just gave up…So it was sort of a loveless relationship but mostly she was unhappy and long story short…I wanted to stay together because she was my only friend and I didn’t want to lose the friendship and the companionship…So I hang on to her for dear life and my proposition was that we try polyamory. We stay together as a household, but she can have a boyfriend and she can have sex and get her needs met sexually…I found her a boyfriend and – yeah, it’s funny because it’s so typically self-sacrificing. I found her a boyfriend and then she left me.”

Charles depicted himself as selflessly forgoing a monogamous relationship to remedy his partner’s unmet sexual needs with a surrogate lover. There was ire and irony mixed in Charles’ narration of himself as *the* victim to his own well-intended remedy. Indeed, Charles’ compensatory efforts rendered him an accomplice, witness and fatality in his partner ending their relationship.

Men’s victim storylines also extended to their experiences of being left. Chuck, a 33-year-old, foregrounded his work challenges as catalyzing ongoing arguments with his wife of 10-years. Victim to working a high-pressure (and high paying) job, Chuck normed the relationship spills and spoils resulting from his work pressures ahead of narrating the unjust tactics used by his partner to end their marriage:

“I knew we were having a rough patch, but the idea of breaking up never entered my head…I felt kind of blindsided because if it had reached that point for her [partner] I would have hoped that she would have said it, and then I would have been like ‘okay, we need to research how to go and do this.’ Instead, she was kind of like planning this whole thing…she like suggested counselling, but…she had no intention of going to counselling to fix the relationship. She was going to counselling to basically break up with me…She had already grabbed a lawyer and everything and already had another plan for a relationship.”

Norming their conflict as benign, Chuck declared himself victim to his partner’s use of counselling to end, rather than save their relationship. He went on to belabour how the lack of warning robbed him of the opportunity to fix their ailing partnership. In essence, Chuck focused on the ‘how’ (rather than the ‘why’) his partner ended their relationship.

For most men the break-up was psychologically injurious, and conflict often escalated after the split. Liam, a 41-year-old father began by telling the story of his wife’s agreement to immigrate to Australia for employment opportunities and a better life. Liam then contrasted a standoff, positioning himself as victim to a “toxic relationship” - a condition emanating from his partner’s upset about, and regret for, the move. Throughout the interview, Liam story-lined provider and protector roles as *his* primary drivers for immigrating – a virtuous masculine project dislocated by his partner dodging border authorities to flee Australia with their children:

“It was clear in her mind what I set out to do [live and work in Australia], she just wasn’t happy being the passenger in the seat of the car anymore and didn’t like where she was being driven, and wanted to get out and that’s what she’s done.”

The victim of a broken promise, Liam contrasted their mutual agreement to immigrate, with the enforced estrangement from his children, and the litigious conflict arising from that situation.

Many participants talked about break-up conflicts relating to child access, job loss, housing issues and legal proceedings. Richard, a 42-year-old father, described spending years and his savings to secure a legal agreement for 50-50 child access after his ex-wife alleged abuse. Tyler, a 64-year-old father, spoke to bereavement, work and housing challenges along with mental health issues as pressure-points in and around the break-up:

“She was trying to buy a new house, another house and it was a bad decision, and I’m sort of saying ‘that’s a really bad decision to try and do this’ and anyway, ‘I’ve got to go and bury my dad’ and while I’m away burying my dad, sort of her response was, ‘Well, I’ve sold our house and you can go where you like,’ in other words, ‘rack off’...that was when I really went downhill because basically it had all just fallen apart. I’d just gone through all this trauma of losing my dad and still just trying to keep the work happening…but living in a caravan out in the bush in the pouring rain and it rained, it rained for a whole year...and so I went deeper and deeper into self-doubt and depression.”

The losses mounted in all that overflowed to Tyler’s break-up. In this context many men saw and depicted themselves as marginalized and enduring significant hardship at the hands of their partner ending the relationship.

In summary, *Victims of circumstance* narratives chronicled men’s ongoing conflicts whereby they were active in the relationship battles, and the break-up induced turmoil. The men’s narratives extended brusque relationship endings with objection masculinity being embodied to contest their marginality in and out of the partnership.

### Narrative 3: accountability and growth

Five men shared *Accountability and growth* narratives describing diverse efforts and mastery for securing positive personal transformations in the aftermath of their partner ending the relationship. These men transitioned *Ill-equipped to stay or to initiate leaving* and *Victims of circumstance* impasse narratives to relate their introspective self-work as rational, necessary and strength-based to make sense of, learn from, and gain some growth in the aftermath of the relationship ending. Accountability involved intensive self-inquiry in which men narrated their efforts for finding, reclaiming, healing and, often-times re-imaging themselves. Their relationships were no less distressing or challenging than others, but the few references that were made to ex-partners were free of blame, often apologetic and even complimentary, with men reckoning their own behaviours and emotions. Mark, a 47-year-old man, foregrounded a tenuous 4-year relationship that “didn’t end well”:

“We got into another argument, she said ‘this is all your fault’, she yelled at me and I cried, and I kind of said to her, ‘don’t say that to me’. She goes, ‘well, the truth hurts doesn’t it’. Then she decided to move out for good, like that’s it, keys and everything. Actually, I was on a business trip and when I came home the apartment was pretty much empty.”

Shifting his briefly told story about the conflict-ridden relationship and the exit of his partner (synonymous with the *Victims of circumstance* narratives) Mark emphasized ownership of his negative behaviours for their anguished partnership:

“I was not in a healthy place. I was in a place of a lot of anger, denial, and a lot of blame. Pretty much after [partner] and I broke up back in 2016 that really catalyzed everything in my journey to this day…we never saw each other face to face to say goodbye, and I collapsed and that was when I really dug deep and started looking up patterns of men in abusive relationships and abusive men tendencies. As painful as it was, I realized I needed to address this [perpetrating abuse], and that’s really what started it all.”

Mark positioned the break-up as an epiphany, “this awakening moment where it was like ‘oh, something is definitely not right with me’, like I’m the recurring pattern here.” His narrative switched between, but also entwined masculine shame and strength. That is, the shame of perpetrating abuse combined with the strength to admit that wrongdoing made available a grand narrative – Mark’s self-work to change his negative injurious behaviours. Mark also offered some explanatory notes for his poor showing in the relationship, “my own personal experiences with depression, anxiety, certainly anger and a lot of trauma…childhood abuse and family violence, an older brother with schizophrenia.” These exposures and challenges were not positioned or claimed by Mark as excusing. Rather, he contextualized them as undealt-with issues that had influenced his wrecking of all his relationships. In essence, Mark narrated a flawed masculine-self, and discordance with the man he wanted to be, as the pivot and push for needing to authentically know, own and change his negative behaviours.

Within these narratives, participants’ retrospections often included work to deconstruct injuries that predated (but infiltrated) the relationship and its demise. Lou, a 61-year-old man, talked about being victim to childhood sexual abuse and negative residential school experiences in mapping his suicide attempt 3-years after his wife ended their 24-year marriage. Layering the traumas, Lou spoke about working on himself, which included examining his domineering style in the relationship:

“I have done a lot of work in the past three years on the first two [sexual abuse and residential school], and just now with the group therapy I am starting to look at questions around my ideals of marriage and love and fatherhood and manhood, so just started working on that end of it all. So I’m fairly comfortable with the childhood sexual abuse and how that affected me, and even the residential school...It wasn’t a very equal partnership [the 24-year marriage]. That little three-year-old boy who was damaged, he actually loved her [partner] more than I did. So, I’ve made that interesting observation and really have explored that and I believe that.”

Squaring away the reach of his childhood traumas, Lou suggested he was currently working to understand his dominance of, and dependence on his ex-partner. Lou’s self-work (and growth) was posited as “in progress” and contingent on him being accountable for his part in the distress and demise of the relationship. In their storylines, Mark and Lou were variably accountable for the effects of their behaviours on partners but wholly committed to deep introspection work to decipher, rationally understand, and improve their behaviours.

Levering growth from such self-work, participants also narrated their efforts to balance responsibilities for the relationship ending. Ian, a 44-year-old father, talked about his role in the demise of his 18-year marriage, a relationship that ended with his partner’s infidelity, and subsequent decision to leave him for the man with whom she’d had the affair:

“I think maybe if I was a bit more emotionally intelligent early on in our relationship some of the things which led my ex to connect with [new partner] may not have been there, I take some responsibility for that. I wish in hindsight that I had assumed my boundaries better than what I did, I know looking back on it, I potentially enabled my ex’s bad behaviour which basically caused like, a lot of internal damage probably between us.”

Ian spoke about his lack of agency for mending a relationship he knew was drifting apart to involve a third party (synonymous with *Ill equipped to stay or to initiate leaving* narratives). On balance, he concluded, “it [the infidelity and break-up] was a lack of compatibility…and obviously I’ve got deficits but that wasn’t the major factor which caused our relationship to end.” While Ian was accountable for what he could have done better in the relationship, he resisted a self-blame narrative to explain (or excuse) his partner’s infidelity. Moreover, Ian talked about how, with the aid of counselling, he’d redirected his energy to focus on the well-being of his children, rather than ruminate about his partner’s infidelity. The storyline signalled Ian’s resolve for, and reinstatement of his masculine protector role:

“The main thing that I learnt [from counselling] was to just change my perspective on things…‘you know, your life is gonna change’ what can you do to try to ensure that the change is sustainable and not goanna be detrimental? I felt once I shifted my focus to making sure the kids are okay, I was kind of okay in that situation. I was still upset but I could just manage the emotions when the kids were around.”

Ian talked about being counselled and coached to be accountable and strategic for when and how he expressed his feelings. Further, he asserted the self-growth that had come with that work, “I think I learnt a lot, I think I became a better dad, hopefully a better parent as well and probably a better boss.” The profits flowing from Ian’s personal growth stood to (eventually) outweigh the losses he incurred with the relationship ending.

Help-seeking was also lauded by participants as critical to securing their self-growth. Carter, a 42-year-old father, foregrounded that for 2-years after his partner ended their 10-year marriage, “I didn’t ask for any help, nothing” because “I was in a state of denial…denying my emotions and denying myself.” Contrasting his maladaptive substance use and resistance to seeking help, Carter told the story of reaching out to build a “support ring” for moving on from his marriage:

“An older man who I met had a unit [bungalow] in the backyard…which he was about to rent out and I remember calling him, saying ‘I need help’. I said ‘I’m moving out and I’m wondering if I can get your place’. That guy went through a separation a couple of years before and I actually asked him to be my mentor through it. So through him I started to get a few lifelines. He showed me some doors, and he said ‘you can open them if you want, you can walk through them if you want’, so one door was ‘here’s a book that I’ve used’, ‘how about writing a journal?’, ‘how about coming with me to a men’s circle?‘, and I was just like ‘yeah I’m going to go through each and every one of these doors because I’m desperate, I have to do it; something has to change’.”

Carter narrated his rock-bottom crisis as the impetus for addressing his pain. Highlighted was his courage to secure a coach, and wisely select from an array of help resources. In detailing the strong fit with his psychologist, Carter provided a commonality for all the effectual help he had received, “another man that I could speak with without fearing judgement or rejection.” Herein, the affirmations and permissions of other men were positioned by Carter as key to being helped, and his subsequent commitment to helping men who experienced similar challenges.

In summary, *Accountability and growth* narratives entailed men highlighting their hard work for self-discovery, reclamation and adjustment. It is important to note that these five participants were middle-aged (four men were in their 40s and one was 61-years-old) fathers, and the complex grief and loss of partner-initiated break-ups were narrated as ongoing gender transformative projects.

## Discussion and conclusion

The current study findings contrast the lock of the two impasse narratives (*Ill equipped to stay or to initiate leaving* and *Victims of circumstance*) with the freeing potential for men to transition those storylines toward *Accountability and growth*. These (and other) impasse narratives might be usefully anticipated and recognized as needing to be interrupted and shifted to reduce the re-injury and escalation potentials that accompany the retelling of, and ruminations about those stories. In what follows, we discuss some empirical and gender theory gains drawn from the current study in advance of making practice recommendations for tailoring interventions for IPV and DV prevention and promoting men’s mental health.

Empirically, the *Ill equipped to stay or to initiate leaving* narrative highlighted how some men characterized themselves as conflict avoidant and positioned their stoic withdrawal as strategic for weathering sub-optimal relationships. The men’s [in]actions, while embodying some idealized masculinities (i.e. emotional stoicism), also forwent masculine ideals for rational, problem-solving to repair ailing partnerships. Herein, participants were stalled, unable to [re]vision or muster healthful relationships. Previous research has called out such silences in relationships as men’s patriarchal power plays for ignoring, controlling and additionally stressing intimate partners (Gottzén, 2019; McQueen, 2017), and though the current study participants did not explicitly recognize or narrate such intensions, some control elements were likely operating within the *Ill equipped to stay or to initiate leaving* narratives. Equally present, however, were indications of some men’s lack of self-esteem and deservingness to express their needs and/or concerns within their relationships. Other possibilities for participants passively staying in deteriorated relationships include loyalty, and penchants for being partnered – values driving masculine resiliencies to norm men’s complicity for being subordinate within (rather than assertive in correcting or ending) distressed partnerships. Irrespective of the underpinnings, there is a curious contrast in the current study findings with longstanding depictions of men as active and controlling agents in heterosexual relationships (Jurva and Lahti, 2019). Men’s subordination also grew in being left by their partner. In essence, relationship challenges and partner-initiated endings were portrayed as happening to men, confirming their shortfalls within the partnership but failing to mobilize their self-work in the aftermath of the break-up. This might be understood as *resignate* masculinity, a failed project where men were left lamenting their deficits – a subordinate position within, and potentially long-after the relationship ended.

The *Victims of circumstance* narrative positioned men as actively engaged with tension-filled relationships, a milieu that prevailed, and often amplified, when partners initiated the break-up. Men’s storylines mapped relational turns and power struggles to contrast the early draw and drivers for the partnership. Men justified the expression of their viewpoints within the relationship as normative, while simultaneously critiquing their partner’s combative nature as transgressing feminine ideals. An array of influencing factors fed the conflict within the relationship – most of which were positioned as residing outside men’s control (and culpability). In essence, the *Victims of circumstance* narrative built the conflict across men’s increasingly tenuous relationships, their unceremonious dumping, and the pangs for dividing assets and/or parenting time after the break-up, to obscure the potential for participants’ self-work and healing. This might be labelled *objection* masculinity – an unending conflictual state fueled by men contesting their marginality within, and long after the break-up.

While we present these two impasse narratives separately, in line with Frank’s (2013) prediction, there is strong potential that they co-exist as agentic defences and explanatory notes for men being stuck in and forced out of distressed relationships. Within this context it is worth reflecting on *resignate* masculinity as conceding failure, and the power drives synonymous with *objection* masculinity as intermixed and inherently challenging in their swing potential. Indeed, the injurious effects of focussing on one or both of these narratives can increase the potential for IPV and DV, as well as men’s mental health challenges (Webermann et al., 2020). While not espousing *Accountability and growth* as a utopian narrative, it is fair to say that these five participants transformed masculine ideals to norm introspective self-work as strength-based and necessarily life changing. These men craved authenticity as a masculine value for their vulnerability and asset-building *Accountability and growth* storylines. Crises and rock-bottom were often foregrounded to justify men’s help-seeking and wise use of external resources. Evident also was temperance for self-blame, wherein men’s life contexts, including adverse childhood events, were courageously narrated traumas to situate and substantiate their self-work as lifelong. Similar to male perpetrators of DV in Gottzen’s (2019) work, while participants’ self-scrutiny of their problem behaviours harnessed accountability and normed help-seeking, the reconstruction of self as a good man was also critically important.

To bridge the two impasse narratives toward *Accountability and growth*, tailored interventions, including narrative therapy, emerge as avenues to help men explore and potentially change their stories to aid recovery, and make improvements for current (and past) relationships (Kross and Ayduk, 2011; Augusta-Scott and Maerz, 2017). Central to this work are efforts for transforming maladaptive masculinities by having men recognize their break-up stories as mirroring cultural conventions that are linked to gender and power discourses (Nylund and Nylund 2003). This is foundational for men advancing impasse narratives to take up masculinities that are free from outmoded, restrictive and stigmatized ideals for being a man. Constructing a narrative of change that makes available positive ways of being in (and out of) relationships (Nylund and Nylund 2003) can drive men’s adaptive self-reflection (Kross and Ayduk, 2011) and psychological adjustment following break-ups (Boals et al., 2011; Capps and Bonanno, 2000).

In recommending tailored interventions such as narrative therapy, we also suggest there are important potential advances for gender theory by integrating masculinities to the design and evaluation of these transformative services (Kågesten and Chandra-Mouli, 2020; Seidler et al., 2022). Connell’s (2005) masculinities framework has usefully offered a plurality of relational gendered scripts within (and after) men’s intimate partner relationships (Khan et al., 2021; Oliffe et al., 2022; River and Flood, 2021). Further, inclusive masculinity theory (IMT), in suggesting that femininity has become less stigmatized to expand the behaviours valued and embodied by men (i.e. increased emotional openness and disclosure), can begin to advance men’s gender equity efforts (Anderson and McCormack, 2018). Within this context, there is strong potential for gendering Giddens’ (2008) view that contemporary egalitarian relationships are characterized by the norming of mutual trust, emotional awareness, and depth of connection. Further, as Frank (2013) notes, Giddens’ concept of the present-day self is an important reflexive project where the postmodern self is responsible for *his* narratives and actions. The current study findings confirm the interlock of agency (narrative revision) and structure (idealized masculinities) to affirm the value for helping men transition the social and self-assigned demoted subordinate and/or marginalized masculinities. That only five participants indicated a redemptive *Accountability and growth* narrative underscores the need to tailor interventions to norm transformative masculinities (Kågesten and Chandra-Mouli, 2020).

Study limitations include the cross-sectional design and reliance on men’s narratives for the findings. To address this, triangulated data sources (i.e., partners) and longitudinal evaluations might highlight shifts over time including emergent perspectives of equitable intimate partner relationships and amicable break-ups. Future studies might also formally examine the acceptability and feasibility of gender transformative interventions aimed at reducing the potential impact of men’s distressed and/or disrupted partnerships.

In conclusion, partner-initiated break-ups are experienced as significant challenges for many men. Herein, men’s narratives are critically important starting points for getting upstream of dire outcomes including DV, IPV and male suicide. To this end, the current study findings affirm the need for men’s tailored relationship programs inclusive of strategies for recognizing, remedying, and growing in and after intimate partnerships (Oliffe et al., 2022).
